# Ectopic Jejunal Variceal Rupture in a Liver Transplant Recipient Successfully Treated With Percutaneous Transhepatic Coil Embolization: A Case Report

**DOI:** 10.1097/MD.0000000000002151

**Published:** 2015-10-30

**Authors:** Satoru Abe, Nobuhisa Akamatsu, Mayumi Hoshikawa, Chikara Shirata, Yoshihiro Sakamoto, Kiyoshi Hasegawa, Norihiro Kokudo

**Affiliations:** From the Department of Surgery, Artificial Organ and Transplantation Division, Graduate School of Medicine, The University of Tokyo, Hongo, Bunkyo-ku, Tokyo, Japan.

## Abstract

Here we present the rupture of ectopic jejunal varices developing in a liver transplant recipient without portal hypertension, which was successfully treated with percutaneous transhepatic coil embolization.

A 48-year-old man with massive melena was admitted to our department. He had undergone liver transplantation for hepatitis B virus-related liver cirrhosis 8 months before, and his postoperative course was satisfactory except for an acute cellular rejection. No evidence of bleeding was detected by upper endoscopy or colonoscopy, but dynamic multidetector computed tomography of the whole abdomen revealed an intestinal varix protruding into the lumen of the jejunum with suspected extravasation. There was no evidence of portal venous stenosis or thrombosis. Immediately upon diagnosis of the ruptured ectopic jejunal varix, percutaneous transhepatic coil embolization was performed, achieving complete hemostasis. The portal venous pressure measured during the procedure was within normal limits. He was discharged from the hospital 11 days after embolization and remained in stable condition without re-bleeding 6 months after discharge.

This is the first report of an ectopic intestinal variceal rupture in an uneventful liver transplant recipient that was successfully treated with interventional percutaneous transhepatic coil embolization. Clinicians encountering liver transplant recipients with melena should be aware of the possibility of late-onset rupture of ectopic varices, even in those having an uneventful post-transplant course without portal hypertension.

## Abstract 2

Supplemental Digital Content is available in the text

## INTRODUCTION

Jejunal varices are uncommon manifestations of portal hypertension and are rarely symptomatic, even in patients with advanced cirrhosis or severe portal hypertension.^1,2^ Bleeding from small bowel varices is reported in 5% of patients with esophageal varices and portal hypertension.^2^

The portosystemic collaterals are thought to shrink or close after liver transplantation because portal hypertension can be relieved by implantation of a normal graft,^3^ yet gastrointestinal bleeding from varices is not an uncommon presentation in recipients after liver transplantation.^4^ It usually occurs, however, in recipients with complications such as graft dysfunction or portal venous thrombosis/stenosis in the early post-transplant period.^5^ Even in such situations, ectopic/solitary jejunal variceal rupture in liver transplant recipients is extremely rare. We herein report a case of ectopic jejunal variceal rupture occurring in an otherwise healthy recipient 8 months after uneventful liver transplantation, which was successfully treated by interventional coil embolization.

## CASE PRESENTATION

A 48-year-old Japanese man with sudden onset melena was admitted to our department. He had undergone deceased-donor liver transplantation with a whole liver graft for hepatitis B virus-related liver cirrhosis 8 months earlier. Before liver transplantation, he had severe portal hypertension with esophageal varices, a spleno-renal shunt, and portal venous thrombus, and massive ascites. During liver transplantation, a thrombectomy was completed, which resulted in an adequate hepatopetal portal flow, and collateral veins remained untouched. His postoperative course was uneventful except for moderate acute cellular rejection on postoperative day 16, which was successfully treated with a steroid bolus and mycophenolate mofetil, and he was discharged on postoperative day 42. Since then, he visited our outpatient clinic for routine post-transplant management and anti-hepatitis B virus combined prophylaxis with entecavir and hepatitis B immunoglobulin. His immunosuppressive regimen included cyclosporine (100 mg twice daily), methylprednisolone (2 mg daily), and mycophenolate mofetil (1500 mg daily).

Upon admission, the patient was slightly tachycardic (heart rate 112/min) with a low systolic blood pressure (78 mm Hg). Physical examination revealed apparent anemia in the conjunctiva, palpation of the spleen 3 cm below the costal margin, and no hepatomegaly. Laboratory data revealed a hemoglobin level of 5.3 g/dL with a slightly decreased platelet count (81 × 10^9^/L), whereas the liver function tests and coagulation panel were within normal limits.

**FIGURE 1:**
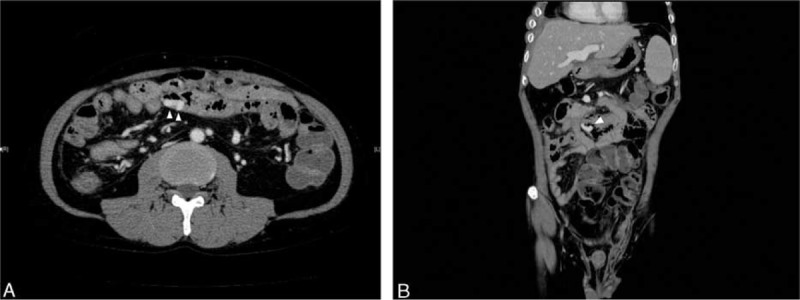
The images of dynamic multidetector computed tomography on admission; the varices protruding into jejunal lumen is pointed by white arrow heads.

After appropriate resuscitation and blood transfusion, the patient underwent an upper endoscopy and colonoscopy, which revealed no evidence of bleeding from gastroesophageal varices, gastroduodenal ulcers, or lesions in the large intestine. At the same time, dynamic multidetector computed tomography (MDCT) of the whole abdomen was performed, which revealed an intestinal varix protruding into the lumen of the jejunum with suspected extravasation (Figure 1A). The patency of the portal venous system without thrombus or stenosis was confirmed. MDCT-angiography revealed that the varix was located at the distal end of the main trunk of the superior mesenteric vein (Figure 1B), which led to our decision to perform immediate interventional treatment rather than double-balloon small-bowel endoscopy or capsule endoscopy.

**FIGURE 2:**
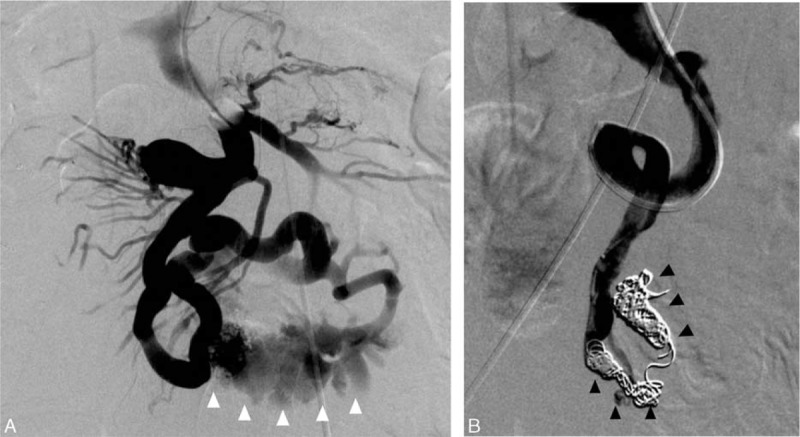
Direct portographies demonstrating variceal rupture (A) and after coil embolization (B); the intraluminal bleeding and coils were pointed by white and black arrow heads, respectively.

Based on the MDCT-angiography findings, the varix was judged to be accessible via a transhepatic approach. Under local anesthesia, portal venous cannulation was performed through left-sided percutaneous transhepatic access using a 21-gauge access needle and 0.018 inch wire, which was then converted to a 0.035-inch system with the placement of a 5Fr sheath. Portal venous pressure was within normal limits (10 mm Hg). Under the guidance of MDCT-angiography, the 5Fr catheter was advanced to the jejunal varix and portography was performed, which revealed rupture of the jejunal varix into the jejunal lumen (Figure 2A and supplemental video, http://links.lww.com/MD/A534). The varix was embolized using 12 metallic coils (4 GDC detachable coils, 8 and 10 mm in diameter, Boston Scientific Japan; 8 Tornado coils, 5–8 mm in diameter, Cook Japan) from the distal to the proximal side. After embolization, complete hemostasis without extravasation was confirmed via completion portography (Figure 2B).

The patient was discharged from the hospital 11 days after the interventional treatment without any rebleeding episodes. Six months after discharge, he remains in stable condition with no further bleeding.

This study was approved by Graduate School of Medicine and Faculty of Medicine, the University of Tokyo Research Ethics Committee/Institutional Review Board (2185). The individual informed consent paperwork was recorded. In the preparation of this manuscript, all efforts have been made to protect patient privacy and anonymity.

## DISCUSSION

Gastroesophageal variceal bleeding commonly presents in patients with portal hypertension, yet jejunal variceal bleeding is rare, even in those with cirrhosis and portal hypertension.^1^ Jejunal variceal bleeding in an uneventful liver transplant recipient is even more uncommon, and, to the best of our knowledge, this is the first report of an ectopic intestinal variceal rupture in such a patient that was successfully treated with interventional coil embolization.

Based on previously published studies,^4–6^ common features of gastrointestinal bleeding after liver transplantation are as follows: it usually occurs within 1 month after transplantation; variceal rupture is often associated with graft dysfunction such as portal venous thrombosis, severe cholestasis, and small-for-size syndrome; Roux-en-Y for hepaticojejunostomy is a frequent site of bleeding; and ulcers and gastroenterocolitis are the major cause of bleeding. No such characteristics applied in the present case.

**FIGURE 3:**
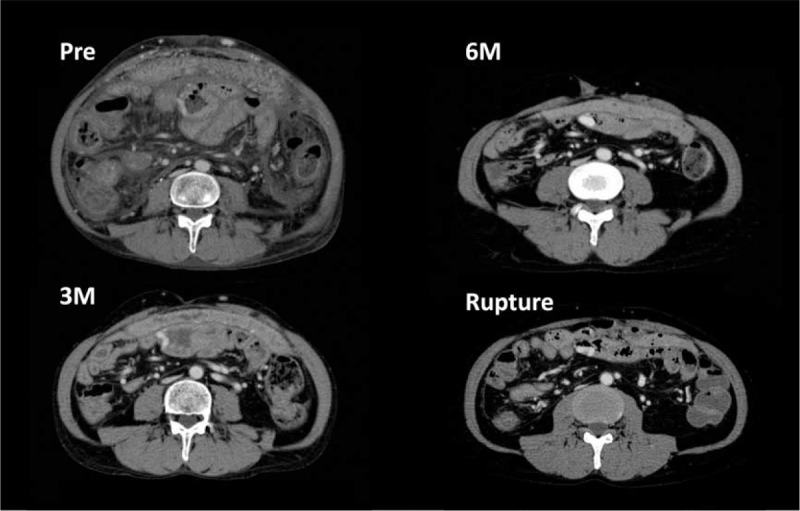
Computed tomography images of the jejunal varices taken at pretransplant, 3 months, 6 months, and readmission.

In liver transplant recipients with an uneventful postoperative course, existing varices usually improve or even disappear with the decompression of portal hypertension.^3^ Indeed, based on a retrospective chronologic review of computed tomography images, the collateral veins observed preoperatively had apparently improved after liver transplantation in this case, but the size of the ruptured varix detected and identified in the pretransplant enhanced-computed tomography remained the same, whereas the intestinal edema had significantly improved (Figure 3). In this particular patient, there was no portal venous thrombus or stenosis, portal venous pressure at the time of interventional treatment was within normal limits, and the estimated volume of the spleen was 432 mL, which was a decrease from 721 mL at the time of liver transplantation, all of which indicated remission and the absence of portal hypertension after liver transplantation. Therefore, although the reason for the rupture is difficult to speculate, we postulated that mechanical intraluminal injury of the mucosa on the protruding varix caused the rupture. Prompt recognition of the bleeding site by MDCT allowed for immediate interventional coil embolization.

The endoscopic approach is an established treatment for esophageal variceal rupture,^7^ but there is no standard direct treatment for ectopic intestinal varices. Traditionally, intestinal variceal bleeding is treated surgically.^8,9^ In some cases, an interventional and indirect strategy, such as a transjugular intrahepatic portosystemic shunt (TIPS)^10^ and balloon-occluded transverse obliteration to decompress portal hypertension have been used to relieve the bleeding.^11–13^ The efficacy of an endoscopic approach seems minimal,^2,14^ although not entirely inadequate.^15^ Successful direct embolizations via a transhepatic approach such as in this case were recently reported by several authors.^16–19^ Macedo et al^20^ reported 14 case series of percutaneous transhepatic embolization for ectopic varices in the gastrointestinal tract, and concluded that the strategy is effective in the short term, but should be followed by a transjugular intrahepatic portosystemic shunt for long-term control of rebleeding. In contrast to the present case, patients suffering from bleeding from ectopic varices were commonly complicated with severe portal hypertension, which makes the surgical option difficult or life threatening. Accordingly, the interventional approach, directly obliterating the bleeding varices via percutaneous transhepatic route^20^ or relieving the high portal pressure via TIPS,^10^ is the first choice for the treatment, which could be done safely for any patient. Surgical intervention may not be indicated for hemostasis, but may be spared for making shunt to decompress the portal hypertension in selected cases.^21^

Treatment for portal hypertension is of utmost importance in patients with risky varices or ruptured varices, but the present case as well as recent reports demonstrate the efficacy of interventional percutaneous transhepatic coil embolization for variceal ruptures of the intestine. Nevertheless, clinicians encountering liver transplant recipients with melena should be aware of the possibility of late-onset rupture of ectopic varices, even in patients with an uneventful post-transplant course without portal hypertension.
